# Application Progress and Prospect of Herbal and Western Medicine Combined with Antiplatelet Therapy for Cardiovascular Events

**DOI:** 10.1155/2021/5563987

**Published:** 2021-07-29

**Authors:** Ya-Ru Ge, Na Huan, Cheng-Long Wang, Pei-Li Wang

**Affiliations:** Center for Cardiovascular Disease, Xiyuan Hospital, China Academy of Chinese Medical Sciences, Beijing 100091, China

## Abstract

Antiplatelet therapy is the key point in the treatment of cardiovascular and cerebrovascular diseases. Effective and safe antiplatelet therapy can avoid the risk of thrombosis or bleeding again. Herbal and Western medicine combined with antiplatelet therapy for ischemic cardiovascular events is a common phenomenon in clinical application, and more and more animal experiments, in vitro cell experiments, and randomized controlled clinical studies have also clarified the efficacy and interaction mechanism of the combination and safety. Herbal and Western medicine combined with antiplatelet therapy has made some progress in improving aspirin resistance and clopidogrel resistance, enhancing antiplatelet and antithrombotic effect, and reducing gastrointestinal adverse reactions caused by antiplatelet drugs. Both of them play the role of antiplatelet and antithrombotic by reducing platelet adhesion, inhibiting platelet activation and aggregation, and inhibiting platelet release, and the combination of drugs is safe. This article elaborates and analyzes the application progress and prospect of Chinese and Western medicine combined with antiplatelet therapy, in order to provide more theoretical support for future research.

## 1. Introduction

According to the data of “Chinese Cardiovascular Health and Disease Report” [1], it is estimated that the number of patients with cardiovascular disease is 330 million, and the death of cardiovascular disease accounts for the first cause of death in urban and rural residents. The burden of cardiovascular disease is increasing day by day, which has become a major public health safety problem in China. Therefore, it is urgent to strengthen the prevention and treatment of cardiovascular disease. Antiplatelet therapy is the focus in the prevention and treatment of cardiovascular thrombotic diseases, and it is also a hot spot in the field of drug research and development. The application of simple antiplatelet therapy of Western medicine not only brings clinical benefits but also brings the risk of thrombosis or bleeding again. How to effectively inhibit thrombosis and avoid bleeding risk and gastrointestinal injury is the focus of antiplatelet therapy in the field of clinical cardiovascular disease. The application of herbal and Western medicine combined with antiplatelet therapy is a common phenomenon in the clinical treatment of ischemic cardio-cerebrovascular diseases at present [2, 3]. The efficacy, safety, and interaction mechanism of the combined application of herbal and Western medicine have also attracted more and more attention [3]. This article elaborates and analyzes the application progress and prospect of Chinese and Western medicine combined with antiplatelet therapy, in order to provide more theoretical support for future research.

## 2. Pharmacokinetic Analysis of Herbal and Western Medicine Combined with Antiplatelet Therapy

With the increasing use of antiplatelet drugs such as aspirin or clopidogrel in combination with herbal or Chinese patent medicine, the study on the pharmacokinetics of the absorption, distribution, metabolism, and excretion characteristics of various active ingredients in drugs in vivo is also increasing [4]. Based on the common clinical combination of drug use, comparative pharmacokinetic studies of Western medicine alone and combined with herbal were carried out, and pharmacokinetic interactions were evaluated by differences in pharmacokinetic parameters. Yujia et al. [5] studied different doses of ginseng stem leaf total saponins interacting with aspirin in rats, and the results found that high doses of ginseng stem leaf total saponins can increase the metabolite of aspirin (600 mg) (salicylic acid) in rats' blood drug concentration, AUC (0-t) and Cmax increased significantly, V is significantly reduced, CL increased significantly, and the relative bioavailability was 1.85 times higher in the aspirin group alone, indicating that total ginseng saponins from stem and leaves can increase the bioavailability of aspirin in vivo and promote the absorption of aspirin in vivo under a certain dose or long-term administration. Some experiments have also shown that total saponins of *Panax notoginseng* and red ginseng decoction can improve the bioavailability of aspirin in vivo [6, 7].

The study of pharmacokinetics of antiplatelet therapy combined with herbal and Western medicine is of great significance to guide rational drug use in clinic. At present, it is generally believed that herbal affects the in vivo process of Western medicine mainly by inducing or inhibiting the activity of drug metabolism enzymes and/or transporters [8]. Zhou [9] researched *Salvia miltiorrhiza* capsule on clopidogrel pharmacodynamics and pharmacokinetics in the human body and the influence of 20 healthy subjects who selected oral dose of clopidogrel and *Salvia miltiorrhiza* capsules and clopidogrel together, and the results found that clopidogrel in the combination group and its active metabolites of pharmacokinetic parameters changed, *Salvia miltiorrhiza* capsule induction of the metabolism of clopidogrel occurred, and the antiplatelet effect of clopidogrel was weakened. At the same time, in vitro study results showed that the two active components (cryptotanshinone and tanshinone IIA) in *Salvia miltiorrhiza* capsule could change the pharmacokinetic parameters of clopidogrel by enhancing the activity of CYP3A4 enzyme and inducing its protein expression, thus exploring the pharmacokinetic and pharmacodynamics mechanism in human body. Xiao et al. [10] found that Gegen combined with Danggui could change the pharmacokinetics of DAPT, including reducing clopidogrel metabolism and reducing the activities of RCYP2C11 and carboxylesterase in rat liver microsomes.

This information is of great significance to the further application of the integration of herbal and Western medicine in clinical practice. The pharmacokinetics of the combination of herbal and Western medicine is worth further clinical verification due to the complexity of the composition of herbal and the difference of varieties.

## 3. The Efficacy Analysis of Herbal and Western Medicine Combined with Antiplatelet Therapy

### 3.1. Improve Antiplatelet Drug Resistance

In the treatment of cardiovascular and cerebrovascular diseases, commonly used antiplatelet drugs include cyclooxygenase inhibitors such as aspirin, adenosine diphosphate (ADP) P2Y12 receptor blockers such as clopidogrel and platelet membrane glycoprotein IIb/IIIa (GP IIb/IIIa) receptor antagonists such as tirofiban. Antiplatelet drugs can inhibit platelet aggregation and antithrombotic effect by acting on a certain target and pathway. However, in clinical practice, some patients may express platelet resistance events after taking antiplatelet drugs. Platelet resistance refers to patients with reduced or no response to antiplatelet drugs, resulting in ischemic cardiovascular and cerebrovascular events, including aspirin resistance and clopidogrel resistance [11]. Studies have found that patients with platelet resistance are more likely to have cardiovascular and cerebrovascular events than patients with platelet sensitivity [12, 13]. At present, there is no specific treatment for platelet resistance. Traditional Chinese medicine has many advantages in the treatment of antiplatelet drug resistance, such as multitarget, multichannel, effectiveness, and safety [14].

Modern pharmacological experiments of Xuefu Zhuyu [15] decoction have proved that it can downregulate the contents of thromboxane A_2_ (TXA_2_) and prostaglandin I_2_ (PGI_2_), reduce platelet adhesion and aggregation, and then inhibit thrombosis. Wu [16] observed the effect of Xuefu Zhuyu decoction on platelet aggregation in patients with aspirin resistance and found that Xuefu Zhuyu decoction combined with aspirin can significantly reduce the platelet aggregation value of aspirin resistance patients with ADP and arachidonic acid (AA) as inducers and improve aspirin resistance, which is a beneficial supplement to aspirin resistance. The main component of Xuesaitong capsule is *Panax notoginseng* saponins. Modern pharmacology has proved that Xuesaitong capsule can block calcium channel, inhibit platelet aggregation, and increase cerebral blood flow [17]. Through the study of Xuesaitong capsule combined with aspirin in the treatment of aspirin resistance in patients with coronary heart disease clinical curative effect, LV [18] found that compared with aspirin combined with dipyridamole, Xuesaitong capsule combined with aspirin for one month can inhibit platelet aggregation, reduce platelet aggregation rate, improve aspirin resistance, reduce thrombosis, and has no adverse reactions and high safety. Ma et al. [19] observed the effect of Xuesaitong combined with clopidogrel on antiplatelet aggregation in male SD rats. The results showed that compared with clopidogrel alone, the combined treatment could significantly reduce the platelet aggregation rate. Clopidogrel alone showed resistance after 42–48 days. The combined treatment could improve the blood cell parameters, blood coagulation parameters, and hemorheology. The synergistic effect showed that the combination of the two drugs could improve the resistance of clopidogrel in long-term medication. Guo et al. [20] observed the effect of Shexiang Baoxin pill on clopidogrel resistance in patients with acute coronary syndrome (ACS) and found that compared with single clopidogrel treatment, Shexiang Baoxin pill combined with clopidogrel treatment can improve the platelet aggregation rate and improve clopidogrel resistance and platelet inhibition rate in patients with ACS. Xie et al. [21], through discussing the clinical value of Tongxinluo Capsule on clopidogrel resistance in ACS patients, found that compared with single clopidogrel treatment, Tongxinluo capsule combined with clopidogrel treatment for 3 months can improve clopidogrel resistance and reduce the incidence of cardiovascular adverse events.

### 3.2. Enhance Antiplatelet and Antithrombotic Effects

Antiplatelet drugs can inhibit platelet adhesion, activation and aggregation, and reduce cardiovascular and cerebrovascular events. The study found that traditional Chinese medicine combined with antiplatelet drugs can enhance the antiplatelet and antithrombotic effect and reduce the incidence of thrombotic events, which has a good clinical prospect [14]. Zhu et al. [22] observed the effect of combined application of compound Danshen tablet and clopidogrel on platelet activation function in hyperlipidemia mice and found that compound Danshen tablet can significantly enhance the effect of clopidogrel on reducing activated platelet ratio, neutrophil platelet aggregation rate, and monocyte platelet aggregation rate in hyperlipidemia mice, and the combination of both can enhance the antiplatelet effect, and it has a good clinical effect. Hong [23] explored the clinical efficacy of compound Danshen dripping pills combined with aspirin in patients with coronary heart disease and found that compared with aspirin alone, the combined application can effectively reduce the maximum platelet aggregation rate (PAGM) and thromboxane B_2_ (TXB_2_) levels and enhance the antiplatelet and antithrombotic effect. Zhang and Zhu [24] observed the efficacy of Shuxuening injection combined with antiplatelet drugs in the treatment of acute cerebral infarction and found that compared with aspirin and clopidogrel dual antiplatelet treatment (DAPT), the combination of Shuxuening injection and DAPT for 15 days can significantly reduce the platelet aggregation rate, plasma viscosity, and hematocrit level, suggesting that Shuxuening combined with DAPT has a synergistic therapeutic effect, which can inhibit platelet activation and aggregation from multiple ways and effectively prevent and treat thrombosis. Chen et al. [25] observed the effect of Buchang Naoxintong capsule (NXT) on patients undergoing percutaneous coronary intervention (PCI). It was found that compared with the DAPT group, NXT combined with DAPT could enhance the antiplatelet effect, and the major adverse cardiovascular events (MACE, including cardiac arrest and acute coronary syndrome) in the combined treatment group were significantly reduced during the 12-month follow-up. Shang et al. [26] carried out a multicenter, randomized, double-blind, placebo-controlled trial. 335 patients after PCI were treated with Xiongshao capsule on the basis of conventional antiplatelet therapy for 6 months. It was found that the combination therapy can safely and effectively reduce the recurrence of angina pectoris and inhibit restenosis in elderly patients with coronary heart disease after PCI, with significant antiplatelet effect.

### 3.3. Reduce the Adverse Reactions of Antiplatelet Drugs

Clinically, the gastrointestinal adverse reactions caused by antiplatelet drugs are very common, such as nausea, vomiting, epigastric discomfort, or pain. Long-term use of antiplatelet drugs is easy to cause gastric mucosal damage, causing gastric ulcer and gastric bleeding [27]. Recent experimental studies have shown that aspirin inhibits platelet activation by inhibiting the synthesis of TXA_2_ and also inhibits the synthesis of PGI_2_, leading to original ulcer bleeding [28]. Clopidogrel inhibits the VEGF-VEGFR-ERK signaling pathway, reduces the expression level of vascular endothelial growth factor (VEGF), inhibits the vascular growth of ulcer tissue, and delays ulcer repair [29]. Kou et al. [30] observed the effect of total saponins of *Panax quinquefolium* (PQS) combined with DAPT on gastric mucosal injury in rats with acute myocardial infarction and found that PQS combined with DAPT can enhance the defense and repair function of gastric mucosal barrier, reduce gastrointestinal erosion and bleeding, and reduce the score of dyspepsia, and its mechanism is related to the upregulated expression of cyclooxygenase-2 (COX-2), prostaglandin E_2_ (PGE_2_), prostaglandin I_2_ (PGI_2_), and VEGF in gastric tissue. Yu et al. [31] found that the combination of Danshensu and aspirin can inhibit the formation of aspirin-induced gastric ulcer, and no gastrointestinal adverse events occurred. This may be attributed to Danshensu's higher selectivity for COX-2, upregulation of 6-keto-prostaglandin F1A, and downregulation of TXB2, normalizing the balance of TXA_2_/PGI_2_. Bai et al. [32] observed the therapeutic effect of fresh juice of *Sedum notoginseng* on gastric hemorrhage model mice established by intragastric administration of aspirin and found that *Sedum notoginseng* can increase the number of platelet and platelet membrane GP IIb/IIIa receptors, promote platelet activation, and have hemostatic effect on gastric hemorrhage caused by aspirin. Other traditional Chinese medicines for promoting blood circulation and removing blood stasis have also been proved to protect gastric mucosal damage caused by antiplatelet drugs [14]. For example, curcumin combined with aspirin can reduce gastric mucosal damage by inhibiting COX-2 activity, reducing intestinal mucosal lipid oxidation and gastric acid secretion [33].

The combination of herbal and Western medicine has significant advantages in improving antiplatelet drug resistance, enhancing antiplatelet and antithrombotic effect, and reducing adverse reactions of antiplatelet drugs, which is the embodiment of the theory of treating the same disease with different methods and treating different diseases with the same method.

## 4. Interaction Mechanism of Herbal and Western Medicine Combined with Antiplatelet Therapy

### 4.1. Decrease Platelet Adhesion

Intact vascular endothelium can inhibit platelet adhesion, so when vascular endothelium is damaged, platelets are activated, leading to the activation of membrane glycoprotein receptor on the surface of platelets. The activated membrane glycoprotein receptor combines with a series of substances, such as von Willebrand factor, fibronectin, and collagen, causing platelets adhere to each other. After platelets adhere to endothelial cells, the morphology of platelets changes. Under the induction of aggregation agents such as ADP, thrombin, platelet activating factor (PAF), collagen, and histamine, a series of biochemical reactions occur, which aggravate the adhesion and aggregation of platelets and eventually lead to thrombosis. Therefore, the protection of vascular endothelial cells and inhibition of the binding of aggregator and receptor are two ways to reduce platelet adhesion.

Ge et al. [34] observed the effect of Maixuekang capsule (MKC) on the long-term prognosis of patients with acute coronary syndrome (ACS) after PCI. The results showed that, after 12 months of treatment, the platelet aggregation rate, serum C-reactive protein, and tissue factor in MKC combined with antiplatelet drugs group were significantly decreased, and the prognosis and quality of life in MKC combined with antiplatelet drugs group were significantly improved. The mechanism of MKC's antiplatelet effect may be related to inhibiting the stimulation of thrombin on endothelial cells which can protect vascular endothelial function, inhibiting the binding of platelet and thrombin and reducing the adhesion of platelet. Wang et al. [35] observed the effect of PQS combined with DAPT on rats with acute myocardial infarction. The results showed that PQS combined with DAPT could protect vascular endothelial function and inhibit platelet aggregation in rats. These effects were caused by PQS regulating the dynamic balance between endothelin (ET) and nitric oxide (NO). Wang et al. [36, 37] have proved that antiplatelet drugs cannot reduce the apoptosis of endothelial cells induced by oxidized low density lipoprotein (ox-LDL), but through PQS or *Panax notoginseng* saponins combined with DAPT treatment, it can raise the PI3K/Akt pathway of endothelial cells, reduce the apoptosis of endothelial cells, and inhibit the platelet adhesion induced by endothelial injury. Lu et al. [38] found that 200 mg/kg *Panax notoginseng* saponins combined with DAPT have protective effects on ox-LDL induced apoptosis of human umbilical vein endothelial cells and can improve endothelial platelet adhesion. Zhou et al. [39] found that the combination of sodium tanshinone IIA sulfonate and clopidogrel in the treatment of patients with coronary heart disease and angina pectoris can more effectively inhibit the secretion of ET and TXB_2_ under the condition of myocardial ischemia, increase the secretion of vascular protective factor NO, and improve vascular endothelial function, which has good clinical efficacy. In a clinical trial involving patients with ACS [40], in addition to conventional Western medicine treatment (aspirin, clopidogrel, statins, percutaneous coronary intervention, etc.), safflower injection combined administration can also improve the clinical symptoms and ECG changes of angina pectoris. Postanalysis showed that the expression of GP IIb/IIIa decreased, suggesting that safflower may also inhibit platelet aggregation.

### 4.2. Inhibition of Platelet Activation and Aggregation

#### 4.2.1. Ca^2+^ Pathway

Intracellular Ca^2+^ concentration is the key to platelet activation. Reducing the concentration of free Ca^2+^ can inhibit platelet activation. Study [41] found that Kok can significantly inhibit the release of adenosine triphosphate (ATP) and the increase of intracellular calcium induced by rat platelet collagen. The mechanism of antiplatelet effect of Kok may involve the inhibition of ATP release and the increase of intracellular calcium.

#### 4.2.2. Arachidonic Acid (AA) Metabolic Pathway

After platelet activation, free AA is transformed into prostaglandin H_2_ (PGH_2_) under the action of COX-1 and COX-2, and the latter synthesizes and releases TXA_2_, PGI_2_, and PGE_2_ under the action of corresponding enzymes. TXA_2_ has a short biological half-life and can be metabolized rapidly to TXB_2_, which can promote vasoconstriction and platelet aggregation; the metabolite of PGI_2_ is 6-keto-PGF1*α*, which can promote local vasodilation and inhibit platelet aggregation. Therefore, the imbalance of TXA_2_/PGI_2_ is one of the important causes of platelet aggregation and thrombosis. Hong [23] explored the clinical efficacy of compound Danshen dripping pills (Danshen, Sanqi, and borneol) combined with aspirin in patients with coronary heart disease and found that the levels of PAGM and TXB_2_ in the combination group were significantly lower. Zou et al. [42] found that the TXB_2_ level of *Trichosanthes kirilowii* combined with the aspirin group was significantly lower, and compared with the aspirin group, TXB_2_/6-keto- PGF1*α* of the combined medium and high-dose groups could be significantly improved and tended to be normal, indicating that the combined therapy can better inhibit platelet activation and aggregation. Kou et al. [30] found that the combination of PQS and DAPT can enhance the antithrombotic effect in rats with acute myocardial infarction. The mechanism may be related to PQS enhancing the inhibition of aspirin on the COX-1/TXA_2_ pathway in platelets and the upregulation of the COX/PGI_2_ pathway and activating the fibrinolytic system.

#### 4.2.3. Cyclic Adenosine Monophosphate (cAMP) Pathway

cAMP and cGMP in platelets are the inhibitory second messengers of various aggregation agents and platelet-specific receptors, which can inhibit platelet aggregation by increasing the concentration of cAMP and cGMP in platelets. Fan et al. [43] studied the antithrombotic mechanism of salvianolic acid A (SAA) and found that SAA (200–1000 g/ml) can increase the cAMP level, which may be mediated by the activation of adenylate cyclase. Its potential mechanism involves the induction of the cAMP pathway, and adenylate cyclase increases the cAMP level to inhibit platelet aggregation and release. Wang et al. [44] explored the effect of Qishen Yiqi dropping pills (QSYQ) on platelet aggregation and its possible mechanism and found that QSYQ combined with aspirin can significantly increase the cAMP level of hyperlipidemia rabbits, suggesting that the mechanism of antiplatelet effect of QSYQ combined with aspirin may be realized through the cAMP pathway.

### 4.3. Inhibition of Platelet Release

After stimulation, platelets activate and release P-selectin (CD62P), serotonin, and AA, which can in turn lead to the release of TXA_2_, TXB_2_, phospholipase C*β*3, phospholipase C*γ*2, the activity of COX-1 and TXA_2_ synthase, and the phosphorylation of proteins. At the same time, these factors in turn promote platelet aggregation [45]. Therefore, inhibiting the release of these substances can play a good antiplatelet effect.

He et al. [46] found that the antithrombotic effect of ligustrazine combined with aspirin and clopidogrel is better than that of aspirin and clopidogrel alone. The combined administration has inhibitory effect on AA, ADP, and PAF, and ligustrazine has the strongest inhibitory effect on platelet aggregation induced by PAF, which indicates ligustrazine combined with aspirin and clopidogrel may have synergistic antiplatelet effect through AA, ADP, and PAF. Tongxinluo capsule is a Chinese patent medicine developed by academician Wu Yiling based on the theory of TCM venation. It is made up of five insect preparations, including scorpion, centipede, leech, cicada, and woodlouse. It assists the use of *Paeonia lactiflora*, artificial borneol, *Panax ginseng*, and other kinds of Chinese medicine, and multiple traditional Chinese medicine combinations play the role of multitarget therapy [21]. Zhang et al. [47] studied the effect of Tongxinluo capsule on patients with acute coronary syndrome after PCI and found that the levels of platelet activating factor acetylhydrolase, ET, and soluble vascular cell adhesion molecule in Tongxinluo capsule combined with the DAPT group were significantly decreased, and the level of NO was increased, which can play the role of antiplatelet.

## 5. Safety Analysis of Herbal and Western Medicine Combined with Antiplatelet Therapy

The combined use of herbal and Western medicine for antiplatelet treatment of cardiovascular events has definite efficacy, but whether the combined use will cause bleeding, aggravation of gastrointestinal damage, and other safety issues have also attracted attention. Therefore, more and more studies have proved the safety of the combined use. Compound Danshen dripping pills are widely used in the treatment of coronary heart disease. Chen et al. [48] applied compound *Salvia miltiorrhiza* dropping pills to clopidogrel-resistant patients and found that compound Danshen dripping pills could further inhibit platelet function, and no bleeding complications such as gastrointestinal bleeding and cerebral hemorrhage occurred, and no liver and kidney function damage was found after treatment. It provides a certain basis for the safety of compound Danshen dripping pills in clinical application. Liu et al. [49] found that the combined application of Xinnaoshutong in patients with aspirin resistance could not only reduce aspirin resistance but also lower the incidence of adverse reactions such as gastrointestinal discomfort, and the safety of the combined application group was higher. It was also found that compared with DAPT group (control group), the incidence of revascularization, recurrent angina pectoris, and bleeding time in DAPT combined with Xuefu Zhuyu Decoction group (combined medication group) were lower than those in the control group. The incidence of adverse reactions in the two groups was not statistically significant, which proved the safety of combined medication.

## 6. Prospect of Herbal and Western Medicine Combined with Antiplatelet Therapy

In recent years, Chinese and Western medicine combined with antiplatelet therapy has made some progress in improving aspirin resistance and clopidogrel resistance, enhancing antiplatelet and antithrombotic effect, and reducing gastrointestinal adverse reactions caused by antiplatelet drugs. Both of them play the role of antiplatelet and antithrombotic by reducing platelet adhesion, inhibiting platelet activation and aggregation, and inhibiting platelet release and the combination of drugs is safe.

Different from the characteristics of modern antiplatelet drugs acting on a single target and single pathway, antiplatelet therapy of traditional Chinese medicine has the characteristics of multicomponent, multitarget, and multilink. Animal experiments and in vitro cell experiments have explored the interaction mechanism of Chinese and Western medicine combined with antiplatelet from many aspects. At the same time, a number of randomized controlled clinical trials [50, 51] around the treatment of Chinese and Western medicine combined with antiplatelet have shown that Chinese medicine can enhance the efficacy of antiplatelet drugs while not increasing the adverse reactions such as bleeding and gastrointestinal discomfort. The above provides reliable experimental and evidence-based medical evidence for the application of Chinese and Western medicine combined with antiplatelet and also arises our thinking: (1) up to now, the research on the interaction mechanism of Chinese and Western medicine combined with antiplatelet mostly stays in the stage of observing intermediate indexes. Whether there is interaction in the signal regulation pathway after platelet activation still needs to be further studied. (2) The existing clinical trials of Chinese and Western medicine combined with antiplatelet therapy have relatively small sample size, short follow-up period, and less observation and research on clinical endpoint events and adverse reaction events. Therefore, large-scale, multicenter, randomized controlled double-blind clinical research is more needed in the future to promote the pace of integration of traditional Chinese medicine and internationalization. (3) Traditional Chinese medicine has multiple targets and links to enhance the antiplatelet and antithrombotic effect, reduce bleeding, gastrointestinal injury, and other adverse reactions and give full play to the advantages of traditional Chinese medicine in syndrome differentiation and treatment and overall regulation. In terms of antiplatelet therapy, traditional Chinese medicine plays a role in supplementing qi and activating blood circulation, nourishing yin and activating blood circulation, promoting blood circulation and removing blood stasis, removing blood stasis, dredging collaterals, and so on. Chinese and Western medicine combined with antiplatelet therapy is of great significance for clinical prevention and treatment of cardiovascular events in the future.
